# Open reading frame correction using splice-switching antisense oligonucleotides for the treatment of cystic fibrosis

**DOI:** 10.1073/pnas.2114886119

**Published:** 2022-01-10

**Authors:** Wren E. Michaels, Cecilia Pena-Rasgado, Rusudan Kotaria, Robert J. Bridges, Michelle L. Hastings

**Affiliations:** ^a^Center for Genetic Diseases, Chicago Medical School, Rosalind Franklin University of Science and Medicine, North Chicago, IL 60064;; ^b^School of Graduate and Postdoctoral Studies, Rosalind Franklin University of Science and Medicine, North Chicago, IL 60064

**Keywords:** antisense oligonucleotides, splicing, nonsense-mediated decay, cystic fibrosis, human bronchial epithelial cells

## Abstract

*CFTR* gene mutations that result in the introduction of premature termination codons (PTCs) are common in cystic fibrosis (CF). This mutation type causes a severe form of the disease, likely because of low *CFTR* messenger RNA (mRNA) expression as a result of nonsense-mediated mRNA decay, as well as the production of a nonfunctional, truncated CFTR protein. Current therapeutics for CF, which target residual protein function, are less effective in patients with these types of mutations due in part to low CFTR protein levels. Splice-switching antisense oligonucleotides (ASOs), designed to induce skipping of exons in order to restore the mRNA open reading frame, have shown therapeutic promise preclinically and clinically for a number of diseases. We hypothesized that ASO-mediated skipping of CFTR exon 23 would recover CFTR activity associated with terminating mutations in the exon, including *CFTR* p.W1282X, the fifth most common mutation in CF. Here, we show that CFTR lacking the amino acids encoding exon 23 is partially functional and responsive to corrector and modulator drugs currently in clinical use. ASO-induced exon 23 skipping rescued CFTR expression and chloride current in primary human bronchial epithelial cells isolated from a homozygote *CFTR-W1282X* patient. These results support the use of ASOs in treating CF patients with *CFTR* class I mutations in exon 23 that result in unstable *CFTR* mRNA and truncations of the CFTR protein.

## Significance

Frameshift and nonsense mutations pose a major problem for disease therapeutic development. Eliminating these mutations from the messenger RNA by inducing exon skipping is a relatively unexplored treatment approach, though it has shown promise for some diseases. Here, we show that eliminating a common stop mutation associated with cystic fibrosis (CF), by inducing the skipping of the exon it is located in, results in a restoration of the open reading frame and recovers CFTR protein function in a manner expected to be therapeutic in CF patients who don’t currently have effective treatment options. These results are an important advancement for the CF community but also have implications for other diseases where terminating mutations are responsible for dysfunction.

Cystic fibrosis (CF) is an autosomal recessive genetic disease caused by mutations in the CF transmembrane conductance regulator (*CFTR*) gene (1). CFTR transports chloride and bicarbonate across the apical surface of epithelial cells. Loss of CFTR expression or function affects multiple organ systems, including the lungs, liver, pancreas, intestines, smooth muscle, and heart (2, 3). In the lung, CFTR-mediated chloride secretion and sodium absorption by the epithelial sodium channel regulate airway surface liquid hydration. Loss of CFTR causes disruption of mucociliary clearance, resulting in the proliferation of airway pathogens, chronic infection, inflammation, and bronchial damage.

Though there are over 2,000 variants in *CFTR*, most therapeutics that are clinically available or in development are designed for specific patient subpopulations with the most common mutations (3, 4). Currently, there are four drugs approved by the US Food and Drug Administration (FDA) that directly target CFTR function. These drugs are referred to as CFTR modulators. Ivacaftor (VX-770) potentiates function of CFTR by increasing the probability of channel opening for gating variants (5–7), and lumacaftor (VX-809) and tezacaftor (VX-661) correct processing and trafficking of CFTR to the cell surface (8–12). A new corrector, elexacaftor (VX-445), works in combination with tezacaftor and ivacaftor (13, 14). While drug development has recently expanded drastically, there is a critical need for therapies to treat patients with rare *CFTR* mutations, in particular, nonsense variants that create premature termination codons (PTCs) resulting in low CFTR expression (4, 15).

One of the most common nonsense mutations associated with CF is *CFTR* p.W1282X (c.3846G > A, *CFTR-W1282X*). *CFTR-W1282X* is the fifth most common CF-causing mutation worldwide and the second most common class I mutation associated with the disease (http://cftr2.org) (16). This mutation results in a truncated CFTR (CFTR_1,281_), removing ∼60% of the nucleotide-binding domain 2 (NBD2) but retaining 1,281 of the 1,480 amino acids in the full-length protein. The truncated CFTR-W1282X protein has processing and/or gating defects but is responsive to potentiator and corrector treatment (17–22). However, *CFTR-W1282X* messenger RNA (mRNA) is degraded by nonsense-mediated mRNA decay (NMD), leading to a decrease in mRNA and protein abundance, thereby limiting the effect of modulator drugs (22, 23). Small-molecule compounds that inhibit NMD have been shown to increase *CFTR-W1282X* expression but, to date, no effective drug candidates targeting NMD have been approved for use (22, 24).

Antisense oligonucleotides (ASOs) are another possible therapeutic approach for treating CF caused by nonsense and frameshift mutations. ASOs are short oligonucleotides chemically modified to create stable, specific, and long-lasting drugs that can be designed to modulate pre-mRNA splicing (25, 26). ASOs can be designed to block splicing and induce skipping of an exon, effectively removing it from the mRNA. This strategy can be useful as a potential therapeutic for *CFTR-W1282X* as amino acid 1,282 resides in exon 23, which is a symmetrical exon wherein the open reading frame phase is the same at both ends of the exon and, thus, can be eliminated from the mRNA without disrupting the *CFTR* open reading frame when the flanking exons are spliced together. This approach would have a therapeutic benefit not only by producing a potentially functional protein isoform with a restored C terminus, as suggested by biochemical and functional studies of CFTR (27–29), but also by eliminating the PTC, stabilizing the mRNA, and increasing protein expression, thereby improving efficacy of CFTR modulators.

Here, we demonstrate that a CFTR isoform lacking the amino acids encoded by exon 23 has partial activity when exposed to CFTR modulator drugs. We identify a splice-switching ASO strategy that induces exon 23 skipping and show that ASO-mediated exon 23 skipping in *CFTR-W1282X* RNA, in both an immortalized human *CFTR-W1282X* bronchial epithelial cell line and primary epithelial cells isolated from a CF patient homozygous for *CFTR-W1282X*, stabilizes the *CFTR* mRNA and recovers CFTR activity. We also provide evidence that ASO-induced exon 23 skipping has partial allele specificity for *CFTR-W1282X*, which could be advantageous in treating CF patients heterozygous for *CFTR-W1282X* and another mutation less responsive to current therapeutics.

## Results

### CFTR mRNA-Lacking Exon 23 Generates a Partially Active Protein.

**Fig. 1.**
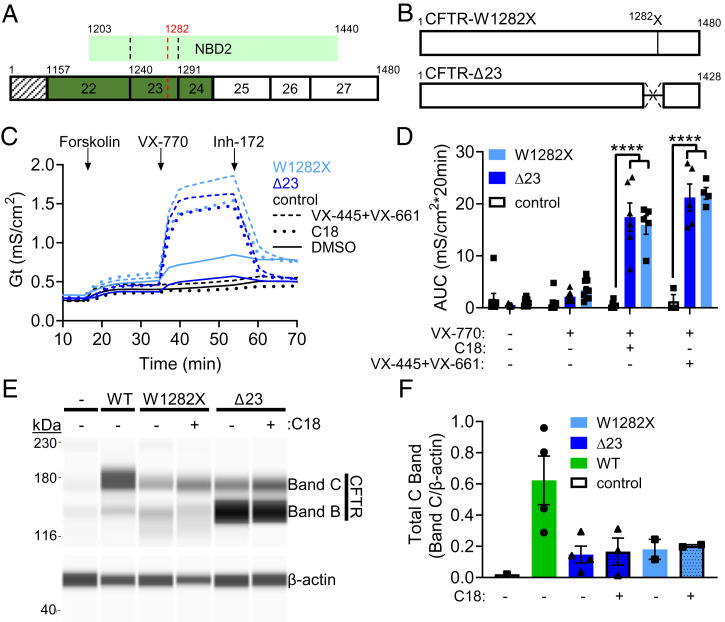
CFTR-Δ23 function is comparable to CFTR-W1282X and is responsive to CF modulators. (*A*) Schematic of CFTR exon 23 in relation to its position within NBD2 and the position of the CFTR-W1282X mutation. Symmetric exons are colored green. (*B*) Schematic of the CFTR-W1282X and CFTR-Δ23 constructs transfected into FRT cells. (*C*) Average conductance traces from FRT cells stably transfected with CFTR-Δ23, CFTR-W1282X, or empty vector. Cells were pretreated with vehicle (DMSO, solid lines), C18 (dotted lines), or VX-445+VX-661 (dashed lines). The time of compound additions (Forskolin, VX-770, or Inh-172) is indicated. (*D*) Average AUC was quantified for the forskolin and VX-770 test periods for each construct. Error bars are ± SEM. Two-way ANOVA; Dunnett’s multiple comparison test to vehicle within groups, ****P < 0.0001. CFTR-Δ23: DMSO and VX-770, *n* = 11; C18, *n* = 6; VX-445+VX-661, *n* = 5. CFTR-W1282X: DMSO and VX-770, *n* = 9; C18, *n* = 5; VX-445+VX-661, *n* = 4. empty vector: DMSO and VX-770, *n* = 8; C18, *n* = 5; VX-445+VX-661, *n* = 3. (*E*) CFTR protein analysis using WES capillary electrophoresis, Bands C and B, isolated from FRT cells stably transfected with empty vector, CFTR-WT, CFTR-W1282X, or CFTR-Δ23 constructs treated with vehicle or C18. β-actin was used as a control for protein expression. (*F*) Quantification of total CFTR C band from *E*, normalized to β-actin. Error bars are ±SEM. Empty vector: *n* = 1, CFTR-WT: *n* = 4, CFTR-W1282X: *n* = 2, CFTR-Δ23: DMSO, *n* = 4; C18, *n* = 3.

The *CFTR-W1282X* mutation resides within exon 23 of *CFTR* RNA. Exon 23 is a symmetrical exon that can be removed without disrupting the open reading frame (Fig. 1*A*). As a first step in determining whether correction of the *CFTR-W1282X* open reading frame by removing exon 23 might be therapeutic, we tested whether an expressed CFTR isoform lacking the amino acids encoded by exon 23 had channel activity (Fig. 1*B*). Fischer rat thyroid (FRT) cells, which lack endogenous expression of cAMP-regulated, apical chloride channels, were transfected with plasmids expressing *CFTR* without exon 23 (CFTR-Δ23) or with the W1282X mutation (CFTR-W1282X). Transepithelial resistance measurements were recorded from the cells after monolayers formed and transepithelial conductance (Gt = 1/Rt) attributed to forskolin-stimulated activation of CFTR and inhibition by CFTR inhibitor, inhibitor-172 (Inh-172), were calculated (30). Measurements were plotted as conductance traces (Fig. 1*C*), and the area under the curve (AUC) was calculated for comparison (Fig. 1*D*). To test the responsiveness of CFTR-W1282X and CFTR-Δ23 to CFTR modulators known to increase CFTR-W1282X function, the cells were treated with VX-770 and C18 (VRT-534), an analog of the corrector VX-809, or VX-445 + VX-661 (8, 10, 13, 31). CFTR-specific activity was undetectable in untreated or VX-770–treated cells. In contrast, both CFTR-Δ23 and CFTR-W1282X had a similar increase in activity following corrector and potentiator treatment (Fig. 1 *C* and *D*). CFTR-W1282X protein appears as faster migrating CFTR Bands C (∼163 kDa) and B (∼138 kDa) below F508del-CFTR and wild-type (WT)–CFTR. CFTR-Δ23 appears as majority Band B (∼147 kDa and ∼170 kDa, Band C), slightly above the W1282X-CFTR Band B, as expected because it retains the C terminus eliminated with CFTR-W1282X (*SI Appendix*, Fig. S2). For both, the increase in functional activity corresponded with the expression of a CFTR protein, as indicated by the presence of the fully glycosylated CFTR isoform (Band C) and the core-glycosylated isoform (Band B) (Fig. 1 *E* and *F* and *SI Appendix*, Fig. S2) (8). These results demonstrate that CFTR-Δ23 has functional activity in the presence of CFTR modulator drugs.

### A Splice-Switching ASO Induces Exon 23 Skipping and Increases *CFTR* mRNA and Chloride-Channel Activity in a *CFTR-W1282X*–Immortalized Human Bronchial Epithelial Cell Line.

**Fig. 2.**
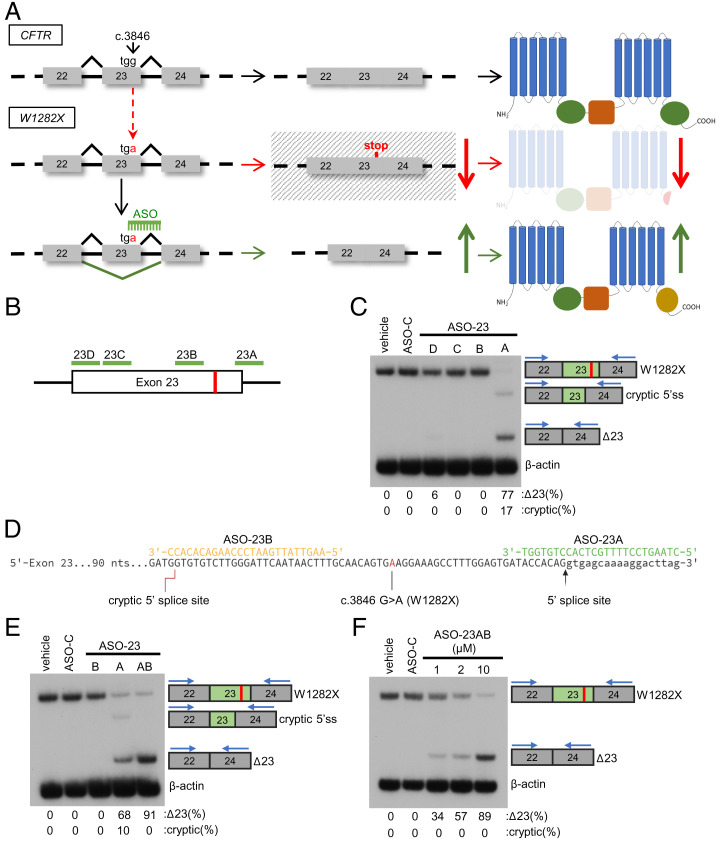
ASOs induce *CFTR-W1282X* exon 23 skipping. (*A*) Schematic of the CFTR dysfunction caused by the *W1282X* mutation in exon 23. The c.3846 G > A (W1282X) mutation creates a PTC in exon 23, leading to degradation of the transcript via NMD and reducing translation of a semifunctional, truncated CFTR protein (indicated by red arrows). ASO induced exon 23 skipping, eliminates the PTC, restores *CFTR* mRNA stability, and increases CFTR expression (green arrows). (*B*) Diagram of ASO target sites (green lines) on human *CFTR* exon 23 pre-mRNA. The location of the *CFTR-W1282X* mutation is indicated by a red vertical line. (*C*) RT-PCR analysis of *CFTR* exon 23 splicing in a CFF16HBEge-W1282X cell line treated with the indicated ASO (10 μM). Exon 23 skipping or cryptic splice site activation was quantified (Δ23 or cryptic/[Δ23+cryptic+W1282X] × 100) and is indicated below each lane. (*D*) Sequence alignment of ASO-23A and ASO-23B to exon 23. (*E*) RT-PCR analysis of exon 23 splicing in CFF16HBEge-W1282X cells treated with ASO-23A, ASO-23B, ASO-23A + ASO-23B (ASO-23AB, 10 μM each), or ASO-C (20 μM). Exon 23 skipping or cryptic splice site activation (percent of total) was quantified and is indicated below each lane. (*F*) RT-PCR analysis of exon 23 splicing in CFF16HBEge-W1282X cells treated with ASO-23AB or ASO-C at indicated concentrations. Exon 23 skipping and cryptic splice site activation was quantified and is indicated below each lane. β-actin is a control for RNA expression in all experiments. Primer-annealing sites are shown above amplicon diagrams.

Splice-switching ASOs are a therapeutic platform that can be used to induce exon 23 skipping to stabilize *CFTR-W1282X* mRNA and increase the abundance of the partially functional CFTR-Δ23 protein isoform (Fig. 2*A*). We tested four ASOs designed to base pair to human *CFTR* exon 23 pre-mRNA and induce exon 23 skipping via a steric block of the splicing machinery (Fig. 2*B*). RT-PCR analysis of CFTR mRNA using primers specific for exons 22 and 24 showed that ASO-23A, which base pairs to the 5′ splice site (Fig. 2 *B* and *D*), induced exon 23 skipping when transfected into an immortalized, patient-derived, bronchial epithelial cell line expressing *CFTR-W1282X* (CFF16HBEge-W1282X) (Fig. 2*C*) (32). ASO-23A also induced splicing at a cryptic 5′ splice site within exon 23, which results in out-of-frame mRNA. To reduce the use of this cryptic splice site and maximize exon 23 skipping, cells were cotransfected with ASO-23A and another ASO, ASO-23B, which blocks the cryptic splice site (Fig. 2*D*). Treatment of cells with ASO-23A and ASO-23B (ASO-23AB) eliminated cryptic splice site use and resulted in a dose-dependent increase in exon 23 skipping (Fig. 2 *E* and *F*).

**Fig. 3.**
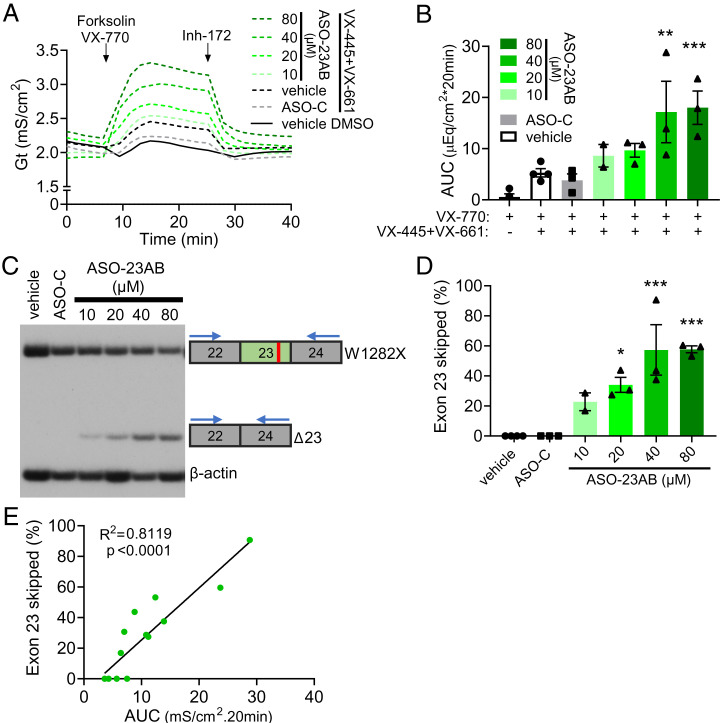
ASO-induced exon skipping increases membrane conductance of a *CFTR-W1282X* cell line. (*A*) Conductance (Gt) traces of a CFF16HBEge-W1282X clonal cell line selected for high resistance (CFF16HBEge-W1282X-SCC:3F2). Cells were transfected with vehicle (black line), ASO-C (gray line), or ASO-23AB (green lines) at indicated concentrations. Cells were pretreated with DMSO (solid line) or VX-445+VX-661 (dashed lines). (*B*) Average AUC of the conductance trace in *A* was quantified for the forskolin + VX-770 test period for each treatment group. Error bars are ±SEM. One-way ANOVA; Dunnett’s multiple comparison test to vehicle + DMSO, **P < 0.01, and ***P < 0.001. *n* = 3, except for 10 μM ASO-23AB in which *n* = 2. (*C*) RT-PCR analysis of exon 23 splicing in CFF16HBEge-W1282X-SCC:3F2 cells in *A*. β-actin is a control for RNA expression. (*D*) Quantification of exon 23 skipping (percent of total) in *C*. Primer-annealing sites are diagrammed. Error bars are ±SEM. One-way ANOVA; Dunnett’s multiple comparison test to vehicle, *P < 0.05, and ***P < 0.001. *n* = 3, except for 10 μM ASO-23AB in which *n* = 2. (*E*) The calculated AUC, shown in *B*, correlated with exon 23 skipping (percent), shown in *D* (simple linear regression).

We next tested whether ASO-23AB treatment could increase conductance in the immortalized hBE *CFTR-W1282X* cell lines. ASO-23AB treatment resulted in a significant increase in conductance when modulators were present compared to controls (Fig. 3 *A* and *B*). The conductance increased in an ASO dose-dependent manner (Fig. 3*B*). This increase in activity corresponded with an increase in exon 23 skipping (Fig. 3 *C* and *D*). There was a positive correlation between ASO-induced exon 23 skipping and conductance (Fig. 3*E*).

### ASO-Induced Exon Skipping Rescues Chloride Currents in Homozygous *CFTR-W1282X* Patient-Derived Bronchial Epithelial Cells.

**Fig. 4.**
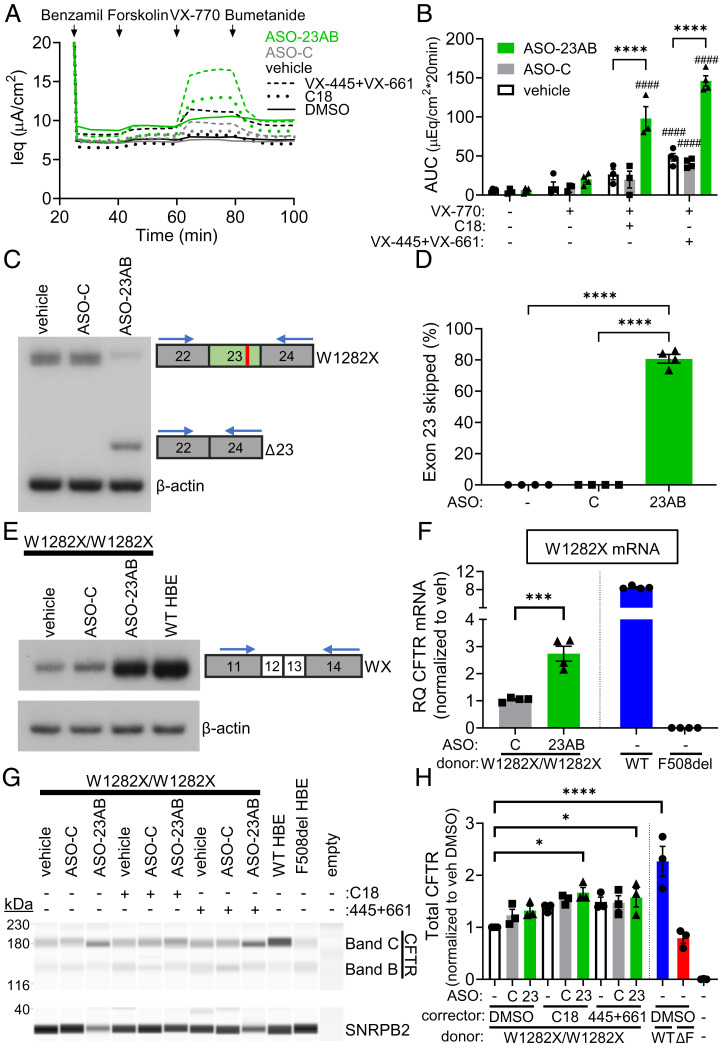
ASO treatment induces exon 23 skipping, stabilizes *CFTR* mRNA, and rescues CFTR function in primary hBE cells isolated from a patient homozygous for *CFTR-W1282X*. (*A*) Equivalent current (Ieq) traces of primary hBE cells from a homozygous *CFTR-W1282X* CF donor. Cells were transfected with vehicle, ASO-C, or ASO-23AB (320 μM total) and pretreated with DMSO, C18, or VX-445+VX-661. (*B*) Average AUC of the current traces in *A* was quantified for the forskolin or forskolin + VX-770 test periods for each treatment group. Error bars are ±SEM. Two-way ANOVA; Dunnett’s multiple comparison test to DMSO within treatment groups, ####*P* < 0.01. Two-way ANOVA; Dunnett’s multiple comparison test to vehicle within treatment groups, ****P < 0.0001. *n* = 4, except C18 treatment in which *n* = 3. (*C*) RT-PCR analysis of exon 23 splicing in cells from *A*. β-actin is a control for RNA expression. (*D*) Quantification of exon 23 skipping in *C*. Error bars are ±SEM. One-way ANOVA; Dunnett’s multiple comparison test, ****P < 0.0001. *n* = 4. (*E*) RT-PCR analysis of *CFTR* mRNA expression (exons 11 to 14) from cells analyzed in *A*, compared to hBE cells from a non-CF donor. (*F*) RT-qPCR analyses of total *CFTR* mRNA (exon 11[WT]-12) from cells analyzed in *A*, compared to hBE cells from non-CF and *F508del-CFTR* donors. Error bars are ±SEM. One-way ANOVA; Tukey’s multiple comparison test, ****P < 0.0001. *n* = 4. (*G*) Capillary electrophoresis (WES) analysis of CFTR protein isolated from cells in *A*. Protein from a non-CF donor and a CF donor homozygous for *CFTR-F508del* is also shown. SNRPB2 is a loading control. (*H*) Quantification of the total CFTR (B + C Bands)/SNRP2 normalized to vehicle + DMSO shown in *G*. Error bars are ±SEM. One-way ANOVA; Dunnett’s multiple comparison test to vehicle + DMSO, *P < 0.05, and ****P < 0.0001. *n* = 3. Primer-annealing sites are shown above amplicon diagrams.

To further assess the therapeutic potential of ASO-induced exon skipping in correcting the *CFTR-W1282X* mutation, we analyzed the effects of ASO treatment on channel activity in differentiated primary human bronchial epithelial (hBE) cells isolated from a CF patient homozygous for *CFTR-W1282X*. The immortalized cell line edited to express the *CFTR-W1282X* mutation is a valuable tool for ASO optimization because of the limited availability of primary cells. However, the hBE cell-based model is the gold standard for preclinical testing of CF therapeutics, as the functional response to drugs in this assay has been shown to accurately predict efficacy in the clinic (33, 34). Transepithelial voltage (Vt) and resistance (Rt) was recorded to calculate an equivalent current (Ieq = Vt/Rt). Without ASO treatment, only the combination treatment of VX-770, VX-445, and VX-661 had a significant effect on chloride secretion in the cells (Fig. 4 *A* and *B*). ASO-23AB treatment in combination with VX-770 + C18, or VX-770 + VX-445 + VX-661, resulted in an approximately fivefold and threefold increase in chloride secretion, respectively, compared to either modulator treatment alone (Fig. 4 *A* and *B*).

The functional rescue by ASO-23AB treatment was accompanied by a significant induction of exon 23 skipping (Fig. 4 *C* and *D*). Quantification of all isoforms of *CFTR* RNA (total CFTR), analyzed using primers to the common exons 11 to 14, revealed that ASO-23AB treatment resulted in a threefold increase in total *CFTR* mRNA compared to untreated samples, a level that is ∼30% of the *CFTR* mRNA quantity in WT non-CF donor hBE cells (Fig. 4 *E* and *F*). The rescue of total *CFTR* RNA expression is indicative of mRNA stabilization, as a result of elimination of the PTC introduced by the *CFTR-W1282X* mutation in exon 23. CFTR-W1282X protein expression was observed in untreated cells from this donor (Fig. 4*G*), possibly resulting from less efficient NMD and likely explaining the low level of chloride secretion when treated with modulators (Fig. 4*B*). In comparison, ASO-23AB treatment resulted in a significant increase in CFTR protein expression (Fig. 4 *G* and *H*). The stabilization of *CFTR-W1282X* mRNA and increase in CFTR protein expression correlates with the robust rescue of chloride secretion in these patient cells, predictive of a potential therapeutic effect of ASO-23AB treatment over current modulator drugs for patients homozygous for *CFTR-W1282X*.

### Activity and Allele Specificity of ASOs in Compound Heterozygous Patient-Derived Bronchial Epithelial Cells.

**Fig. 5.**
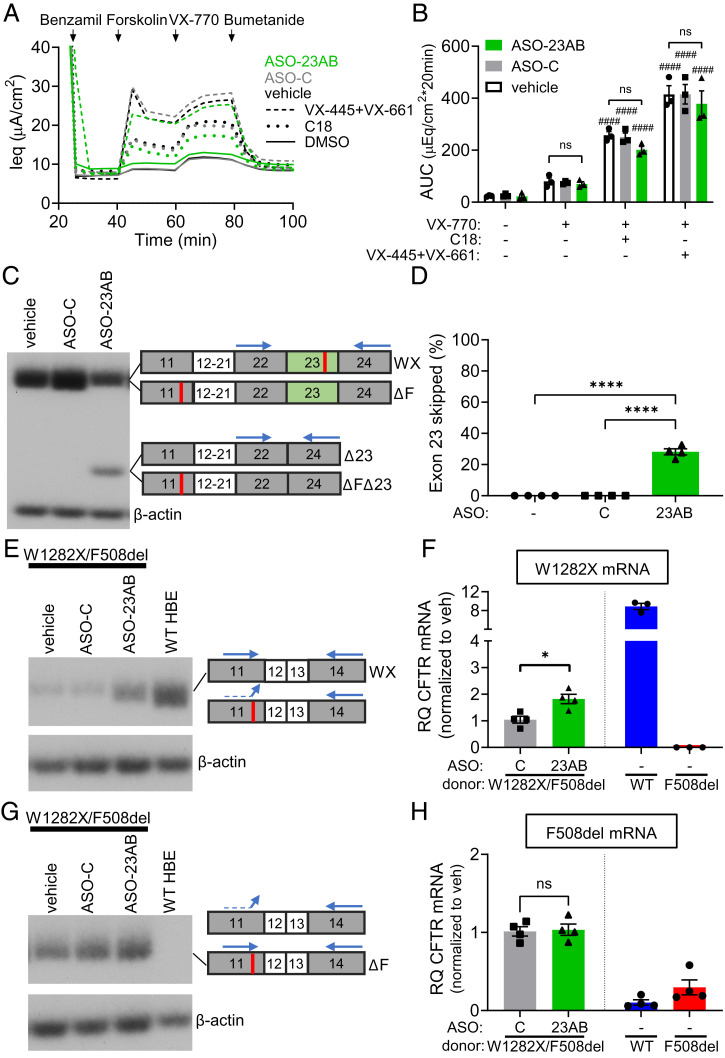
ASO treatment does not affect modulator activity in hBE cells isolated from a CF patient compound heterozygous for *CFTR-W1282X* and *F508del*. (*A*) Equivalent current (Ieq) traces of primary hBE cells isolated from a CF donor heterozygous for *CFTR-W1282X* and *F508del*. Cells were transfected with vehicle, ASO-C, or ASO-23AB (320 μM total). Cells were pretreated with DMSO, C18, or VX-445+VX-661. (*B*) Average AUC of the current traces in *A* was quantified for the forskolin + VX-770 test periods for each treatment group. Error bars are ±SEM. Two-way ANOVA; Dunnett’s multiple comparison test to DMSO within treatment groups, #*P* < 0.05, ####*P* < 0.01. Two-way ANOVA; Dunnett’s multiple comparison test to vehicle within treatment groups, ns = *P* > 0.05. *n* = 3. (*C*) RT-PCR analysis of exon 23 splicing in hBE *CFTR-W1282X/F508del* cells in *A*. β-actin is a control for RNA expression. (*D*) Quantification of exon 23 skipping in *C*. Error bars are ±SEM. One-way ANOVA; Dunnett’s multiple comparison test, **P < 0.01. *n* = 3. (*E*) RT-PCR analysis of non-F508del *CFTR* mRNA expression (exons 11WT-14) from cells analyzed in *A*, compared to hBE cells from a non-CF donor. (*F*) RT-qPCR analyses of total *CFTR* mRNA from non-F508del alleles (exons 11WT-12) in cells analyzed in *A*, compared to hBE cells from non-CF and *F508del-CFTR* donors. Error bars are ±SEM. One-way ANOVA; Tukey’s multiple comparison test, ****P < 0.0001. *n* = 4, one outlier identified and removed with ROUT outlier analysis (Q = 5%) in WT. (*G*) RT-PCR analysis of *CFTR-F508del* mRNA expression (exons 11F508del-14) from cells analyzed in *A*, compared to hBE cells from a non-CF donor. (*H*) RT-qPCR analyses of total *CFTR* mRNA from F508del alleles (exons 11WT-12) in cells analyzed in *A*, compared to hBE cells from non-CF and *F508del-CFTR* donors. Error bars are ±SEM. One-way ANOVA; Tukey’s multiple comparison test, ns = *P* > 0.05. *n* = 4. Primer-annealing sites are shown above amplicon diagrams; the dashed line indicates a mismatch.

Many CF patients with the *CFTR-W1282X* mutation are compound heterozygotes with a different mutation, most commonly *CFTR-F508del*, in the other *CFTR* allele. This second allele would also be a target of ASO-induced exon 23 skipping and would result in a CFTR protein with the original mutation and a deletion of exon 23. To test the effect of ASO-induced exon 23 skipping on mRNA from *CFTR* mutations commonly found with *CFTR-W1282X* in compound heterozygotes, we treated primary hBE cells isolated from a CF patient, with the *CFTR-W1282X* and *CFTR-F508del* mutations, with ASO-23AB and measured chloride secretion (Fig. 5*A*).

In cells from this patient, both modulator combinations of VX-770 and C18 or VX-770, VX-445, and VX-661 resulted in significant recovery of chloride secretion, as expected given that *CFTR-F508del* is known to be responsive to each drug (Fig. 5*B*). ASO-23AB treatment had no significant effect on this rescue, with cells showing no increase or decrease in potentiator and corrector response (Fig. 5*B*).

Analysis of *CFTR* RNA splicing revealed a significant induction of exon 23 skipping with ASO treatment compared to the controls. However, exon 23 skipping was considerably lower (25% of total RNA) (Fig. 5 *C* and *D*) than the skipping obtained in *CFTR-W1282X* homozygous donor cells (80% of total RNA) (Fig. 4 *C* and *D*). These results suggest that exon 23 skipping of RNA from *CFTR-F508del* may be less efficient.

To analyze the effect of ASO-23AB on exon 23 skipping in *CFTR* mRNA derived from each allele specifically, primers were designed to anneal at the *CFTR-F508del* mutation site in exon 11 and specifically amplify either *non-F508del* or *F508del* mRNA (Fig. 5 *E* and *G* and *SI Appendix*, Table S1). When comparing the baseline expression of mRNA from each allele, RNA derived from the *CFTR-W1282X* allele was only 20% of that expressed from the *CFTR-F508del* allele (*SI Appendix*, Fig. S3*A*), most likely because of transcript degradation by nonsense-mediated decay. ASO treatment significantly increased this expression to ∼40% of that generated from the *CFTR-F508del* allele (*SI Appendix*, Fig. S3*A*). Analysis of RNA from each allele separately revealed a twofold increase in *CFTR* transcripts from *CFTR-W1282X*, similar to levels achieved in the homozygous donor and up to 20% of WT *CFTR* expression (Fig. 5 *E* and *F*). In contrast, the ASO had no significant effect on total *CFTR* mRNA expression from the *CFTR-F508del* allele (Fig. 5 *G* and *H*).

To further investigate the reason for an increase in total mRNA from the *CFTR-W1282X* allele following ASO treatment yet an overall reduction in total exon 23 skipping compared to the homozygous donor cells, we analyzed ASO-induced exon 23 skipping from each allele using allele-specific primers to amplify either total CFTR transcribed from *W1282X-CFTR* or *F508del-CFTR*, followed by nested PCR analysis of exon 22 to 24 splicing (*SI Appendix*, Fig. S3 *B*, *Top*). This allele-specific analysis of exon 23 skipping revealed robust exon skipping in mRNA from the *CFTR-W1282X* allele (71% skipping) compared to mRNA from the *CFTR-F508del* allele (2% skipping), suggesting that ASO treatment has a greater effect on splicing of *CFTR-W1282X* exon 23 than on *CFTR-F508del* (*SI Appendix*, Fig. S3*B*).

Comparative sequence analysis of exon 23 indicated that binding sites for several splicing proteins are eliminated by the G > A change in *CFTR-W1282X* compared to WT *CFTR* (ESEfinder3.0) (*SI Appendix*, Fig. S3*C*) (35). The elimination of these splicing enhancer cis-acting sequences may weaken splicing to the exon and make the ASO more effective at inducing skipping. In fact, ASO treatment in non-CF and *CFTR-F508del* homozygous donor cells in comparison to donor cells with either one or two copies of *CFTR-W1282X* showed an increase in ASO-induced exon 23 skipping that correlated with the number of *CFTR-W1282X* alleles (*SI Appendix*, Fig. S3*D*). We did not assess the effects of nonsense-mediated decay in cells expressing the *CFTR-W1282X* stop mutation, which may artificially increase the exon-skipped isoform relative to the exon-included isoform. Nonetheless, these results, combined with the analysis of total CFTR mRNA (Figs. 4*F* and 5*F*), suggest that ASO-23AB may be more effective at inducing exon 23 skipping in *CFTR-W1282X* RNA, which could be advantageous in treating CF patients compound heterozygous for *CFTR-W1282X* and another *CFTR* mutation less responsive to modulator treatment.

## Discussion

Despite clinical success, the use of ASOs to correct the translational open reading frame and recover gene expression in diseases, caused by frameshift or nonsense mutations resulting in PTCs, has not been extensively explored as a therapeutic approach. These types of mutations are the most common, disease-causing mutations and account for ∼20% of disease-associated mutations in CF. Here, we demonstrate that elimination of the relatively common nonsense mutation, *CFTR-W1282X*, by ASO-induced skipping of *CFTR* exon 23, which encodes the mutation, recovers CFTR expression. The activity of this CFTR isoform, lacking 52 amino acids, requires CFTR modulator drugs that are currently used to treat CF patients. Thus, this ASO approach in combination with current CF drugs offers a potential therapeutic for individuals with the *CFTR-W1282X* mutation and opens the door for similar strategies to treat other terminating mutations, both in CF and other diseases.

Gene mutations that result in PTCs are challenging to treat because they not only encode a truncated protein product but the mRNA intermediate is a target of NMD, a cellular quality control mechanism whereby mRNA with PTCs are degraded (36, 37). Studies have shown that, if produced at sufficient levels, CFTR-W1282X protein is responsive to current CFTR modulator therapies (17, 19, 20, 22, 23), but because stop mutations in CFTR result in a dramatic loss of *CFTR* expression, CF patients are not usually responsive to these drugs. Approaches are being pursued to identify molecules that stabilize mRNA by blocking NMD but, because NMD is an important mechanism-regulating gene expression, any approach must avoid global inhibition of NMD, which would likely have toxic effects (22, 24). Small molecules that promote translational readthrough of termination codons are also being explored as potential treatments for PTC mutations, including CF (38–43). However, effects from the long-term use of these drugs have raised concerns (42, 44, 45). Though these approaches may hold promise, none are specific to *CFTR* directly, and to date, none have been approved for use in CF patients. More recently, gene-specific suppression of NMD has been explored as a promising approach to overcome potential risk of global NMD knockdown (46, 47).

Using ASOs to remove exons encoding stop codons to correct the translational open reading frame has broad applications for addressing terminating mutations. ASO-mediated reading frame correction via induced skipping of symmetrical exons has shown promise in the FDA-approved ASOs targeting PTCs in Duchenne’s muscular dystrophy (48) and also in preclinical studies in mice for the treatment of diseases such as CLN3 Batten (49). A critical requirement for this approach is that the induced protein isoform must retain partial function. We show that CFTR-Δ23 had significant, cAMP-activated conductance responses that were further enhanced by modulator treatments (Fig. 1). Exon 23 encodes amino acids near the C terminus of CFTR, including a portion of the NBD2. The retained function of CFTR-Δ23 is consistent with previous reports that truncation at NBD2 results in a CFTR protein that is trafficked to the cell surface, albeit with deleterious effects on channel gating (27, 29). The result also aligns with data showing that the *CFTR-W1282X* mutation is responsive to modulator therapies (17–19) (Fig. 1). Notably, though truncation of CFTR at NBD2 results in some retained function, domains at the C terminus are important for stability and gating, and these domains are preserved when exon 23 is skipped (*SI Appendix*, Fig. S2) (28, 29, 50, 51).

The clinical potential of ASO delivery to the respiratory system, one of the primary targets for CF therapeutics, has been demonstrated for asthma and other inflammatory lung conditions (52–55). Naked ASOs have been successfully delivered to the lung, where they access multiple cell types, including cells which express *CFTR* (53, 56–58). Aerosolization of ASOs have shown promise in delivery in both a CF-like lung disease model in mice as well as CF patients (57, 59, 60). Additionally, ASOs have been shown to have long-lasting effects in primary hBE cells isolated from CF patients (61) and in vivo, with treatment durations lasting weeks to months before further dosing is needed in patients (25, 62–64). In addition, as patients with class I mutations tend to have disease phenotypes in other organs, including the digestive system, systemic delivery of ASOs may also be considered (65, 66). Intravenous and subcutaneous injections of ASOs are currently used to treat patients, and bioavailable ASO formulations that target the gut epithelia have shown some potential (67–69).

Unlike other therapeutics targeting global NMD or inducing translational readthrough, our ASO targets *CFTR* transcripts specifically to induce skipping of exon 23 (Fig. 2). ASO treatment in immortalized and patient-derived hBE cells expressing *CFTR-W1282X* resulted in a dose-dependent increase in exon 23 skipping that correlated with an increase in function (Figs. 3 and 4). Furthermore, assessment of mRNA from ASO-treated primary hBE cells from a patient with *CFTR-W1282X* and *CFTR-F508del* revealed partial allele specificity of ASO-induced exon 23 skipping for *CFTR-W1282X* (*SI Appendix*, Fig. S3), potentially broadening the scope of this ASO strategy to CF patients heterozygous for *CFTR-W1282X* and another CFTR mutation less responsive to current modulator therapies. Despite effectively inducing skipping of *CFTR-W1282X* mRNA, ASO-induced exon 23 skipping did not have an improved effect on function compared to modulator treatment alone (Fig. 5). It is possible that a functional ceiling is achieved with modulator treatment in cells from this compound heterozygous donor, and additional expression from the *CFTR-W1282X* allele does not increase chloride current above what was already achieved with modulator rescue on *CFTR-F508del*, for which the modulators were developed. There have been a number of studies identifying other corrector and potentiator drugs superior to these modulators in the context of *CFTR-W1282X* (17, 20, 22, 70). The effect of these new modulators was not tested along with ASO treatment here, but they have been shown to be effective in rescuing *CFTR-W1282X* activity in conjunction with other readthrough compounds that enhance *CFTR-W1282X* expression (22, 70). Future studies may reveal better modulator/ASO combinations for treating *CFTR-W1282X* compound heterozygotes. Overall, our results support the use of ASO treatment in combination with approved CF modulators as an effective treatment option for CF patients with class I mutations within symmetrical exons.

## Materials and Methods

### Expression Plasmids.

CFTR-Δ23 and CFTR-W1282X were created from the synthetic *CFTR* high-codon adaption index construct subcloned in the pcDNA3.1/Neo(+) vector (71) using the Q5 site-directed mutagenesis kit (New England Biolabs [NEB]) with primers flanking each exon (*SI Appendix*, Table S1). All plasmids were sequenced to confirm mutations. Plasmids were stably transfected into FRT cells in 6-well plates using lipofectamine LTX (Thermo Fisher Scientific) and OptiMEM (Thermo Fisher Scientific) for 48 h. Cells were transferred to T75 flasks, and clonal cell lines were selected with G418 (300 μg/mL) for 1 wk. After selection, cells were maintained in media supplemented with G418 (150 μg/mL).

### Cells and Culture Conditions.

FRT cell lines were cultured in F12 Coon’s modification media (Sigma, F6636) supplemented with 10% fetal bovine serum (FBS) and 1% penicillin-streptomycin (PenStrep). 16hBEge-W1282X cell lines were obtained from the Cystic Fibrosis Foundation (CFF) and cultured according to their instructions in minimum essential medium (MEM) media (32, 72). Single-cell clones of the original CFF16hBEge-W1282X cell line were created to select for high-resistance clonal cell lines. One clonal cell line, CFF16hBEge-W1282X-SCC:3F2, was selected for analysis. Primary hBE cells isolated from CF patients homozygous for *CFTR-W1282X* (patient code HBEU10014) and compound heterozygous for *CFTR-W1282X* and *CFTR-F508del* (patient code HBEND12112) were also obtained from the CFF. For functional analysis, cells were differentiated by plating on Costar 24-well high-throughput screening filter plates (0.4 μM pore size, Polyester, Corning, catalog No. CLS3397). FRT and 16hBE cells were grown in a liquid/liquid interface (180 μL apical/700 μL basolateral) in a 37 °C incubator with 90% humidity and 5% CO_2_ for 1 wk. Primary hBE cells were differentiated in an air/liquid interface for 5 wk. Media was replaced three times a week.

### Antisense Oligonucleotides.

Splice-switching ASOs are 25-mer phosphorodiamidate morpholino oligomers (Gene-Tools, LLC) (*SI Appendix*, Table S1). A nontargeting phosphorodiamidate morpholino oligomer (PMO) was used as a negative control, ASO-C, (Gene Tools, standard control oligo). ASOs were formulated in sterile water. ASOs were evaluated for potential off-target effects by basic local alignment tool (BLAST) analysis using ASO-23A or ASO-23B sequences. Targets with potential for base pairing with 15 or more contiguous nucleotides of the ASOs are listed in *SI Appendix*, Fig. S1*A*. No targets with more than 16 contiguous nucleotides of either ASO were found. Targets within exons or near splice sites (within 200 nucleotides) were tested for altered splicing by RT-PCR analysis using primers annealing in flanking exons. No aberrant mRNA products indicative of off-target ASO binding were found (*SI Appendix*, Fig. S1*B*).

### ASO Cell Transfection.

For splicing analysis and ASO optimization outlined in Fig. 2, CFF16hBEge-W1282X-SCC:3F2 clones were transfected with ASOs at indicated concentrations on 24-well plates in MEM media supplemented with 10% FBS and 1% PenStrep. Cells were transfected using Endo-Porter (Gene-Tools, 6 μL/mL) for 48 h before RNA extraction and analysis (73).

For functional analysis outlined in Fig. 3, CFF16HBEge-W1282X-SCC:3F2 cells were transfected on filter plates 4 d postplating. Cells were transfected with ASOs apically in 100 μL complete MEM media, with Endo-Porter at indicated concentrations for 48 h. Transfection reagents were removed before corrector treatment and functional analysis.

Primary hBE cells were transfected after differentiation on filter plates, as previously described (61). Briefly, cells were transfected with ASO in an apical, hypoosmotic solution for 1 h. The solution was removed, and the cells were treated again in Dulbecco's phosphate-buffered saline (DPBS) for 4 d until functional analysis.

### RNA Isolation and RT-PCR.

RNA was extracted from cells using TRIzol, according to manufacturer instructions (Thermo Fisher Scientific). Reverse transcription was performed on total RNA using the GoScript Reverse Transcription System with an oligo-dT primer (Promega). Splicing was analyzed by radiolabeled PCR of resulting complementary DNA (cDNA) using GoTaq Green (Promega) spiked with α-^32^P-deoxycytidine triphosphate. Primers for amplification are reported in *SI Appendix*, Table S1. Reaction products were run on a 6% nondenaturing polyacrylamide gel and quantified using a Typhoon 7000 phosphorimager (GE Healthcare) and ImageJ software (74).

### Real-Time qPCR.

Real-time qPCR was performed with PrimeTime Gene Expression Master Mix and PrimeTime qPCR probe assay kits human non-F508del-CFTR (Integrated DNA Technologies [IDT]; qhCFTR-ex11WTF, qhCFTR-ex12WTR, and hCFTR-F508) and human F508del-CFTR (IDT; qhCFTR-ex11ΔFF, qhCFTR-ex12ΔFR, and hCFTR-DF508) transcripts were normalized to human HPRT1 (IDT; Hs.PT.58v.45621572) (*SI Appendix*, Table S1). All reactions were analyzed in triplicate on 96-well plates and averaged together to comprise one replicate. Real-time PCR was performed on an Applied Biosystems ViiA 7 Real-Time PCR System. Results were analyzed by the ΔΔCT method (75).

### Protein Isolation and Automated Western Blot and Immunoblot Analysis.

Following functional analysis, cell lysates were prepared using Nonidet P-40 lysis buffer (1.0% Igepal, 150 mM NaCl, and 50 mM Tris⋅HCl pH 7.6) supplemented with 1× protease inhibitor mixture (Sigma-Aldrich, catalog No. 11836170001). Protein concentration was measured using a Coomassie (Bradford) protein assay (Thermo Fisher Scientific, catalog No. 23200). Cell lysates were prepared using the sample preparation kit (Protein Simple) for an automated capillary Western blot system, WES System (Protein Simple) (76, 77). Cell lysates were mixed with 0.1× sample buffer and 5× fluorescent master mix for a final protein lysate concentration of 0.2 mg/mL (FRTs) or 1.5 mg/mL (hBEs). Samples were incubated at room temperature for 20 min and then combined with biotinylated, protein-size markers; primary antibodies against CFTR (Ab-432 for FRT lysates; Ab-570 + 450 for hBEs) (from John Riordan, University of North Carolina at Chapel Hill [UNC], Chapel Hill, NC, CFF, diluted 1:100 or 1:50 + 1:200, with milk-free antibody diluent); β-actin (C4, Santa Cruz Biotechnology, sc-47778, diluted 1:50 with milk-free antibody diluent); SNRBP2 (4G3, B’’, provided by Dr. Adrian Krainer and diluted 1:2,000 with milk-free antibody diluent); horseradish peroxidase (HRP)–conjugated secondary antibodies; chemiluminescence substrate; and wash buffer and dispensed into respective wells of the assay plate and placed in WES apparatus. Samples were run in duplicate or triplicate. Signal intensity (area) of the protein was normalized to the peak area of the loading control C4, β-actin (FRT), or B’’, SNRPB2 (hBE). Quantitative analysis of the CFTR B and C Bands was performed using Compass software (Protein Simple).

For traditional immunoblot analysis, cell lysates were similarly isolated and prepared for analysis by dilution to 1.5 mg/mL with NuPAGE™ lithium dodecyl sulfate (LDS) 4× sample buffer (Invitrogen, NP0007). Lysates were separated by 4 to 12% sodium dodecyl sulfate–polyacrylamide gel electrophoresis and transferred to 0.45 μm polyvinylidene difluoride membranes. Membranes were blocked in a 5% nonfat dry milk solution for 1 h, and primary antibodies against CFTR (Ab-432; diluted 1:1,000) or β-actin (C4, 1:2,000) were diluted in blocking solution and incubated with membranes at 4 °C overnight. HRP-conjugated antibodies were diluted in blocking solution and incubated with membranes at 4 °C for 1 h, and protein was detected via chemiluminescence.

### Automated Conductance and Equivalent Current Assay.

Stably transfected FRT cells, CFF16hBEge-W1282X-SCC:3F2, and primary hBE cells were treated with C18 (6 μM) (VRT-534, VX-809 analog), VX-445 + VX-661 (3 μM + 3.5 μM FRT, 1 μM + 3 μM 16hBE, and primary hBE) or vehicle (equivalent DMSO) at 37 °C (31). Around 24 h later, the cells were switched from differentiation media to Hepes-buffered (pH 7.4) F12 Coon’s modification media (Sigma, F6636), apically and basolaterally, and allowed to equilibrate for 1 h at 37 °C without CO_2_. To obtain the conductance measurements, the Rt was recorded at 37 °C with a 24-channel transepithelial current clamp (TECC) robotic system (EP Design), as previously described (78). Briefly, for the FRT cells, baseline measurements were taken for ∼20 min. Forskolin (10 μM) was first added to the apical and basolateral sides, and then, cells were treated with potentiator, VX-770 (1 μM). Finally, Inh-172 (20 μM) was added to inactivate CFTR. The CFF16hBEge-W1812X-SCC:3F2 clones were measured similarly apart from forskolin and VX-770 added to the cells at the same time. Measurements were taken at 2-min intervals. Gt was calculated by the reciprocal of the recorded Rt (Gt = 1/Rt), after Rt was corrected for solution resistance (Rs) and plotted as conductance traces (Figs. 1*C* and 3*A*). Calculated equivalent currents (Ieq) were obtained similarly and as outlined in Michaels et al. (61). Ieq was calculated using Ohm’s law (Ieq = Vt/Rt) and plotted as current traces (Figs. 4*A* and 5*A*). To estimate average functional response trajectories during each test period, AUC measurements of forskolin and forskolin + VX-770 were calculated using a one-third trapezoidal rule for each test period using Excel. The average of two identically treated wells was calculated for each plate to obtain one biological replicate used in the final mean ± SEM graphed.

### Statistics.

Statistical analyses were performed using GraphPad PRISM 9.2.0. The specific statistical test used in each experiment can be found in the figure legends.

## Supplementary Material

Supplementary File

## Data Availability

All study data are included in the article and/or *SI Appendix*.
