# Supplementary material for: Open reading frame correction using splice-switching antisense oligonucleotides for the treatment of cystic fibrosis
Source: Proc Natl Acad Sci U S A. 2022 Jan 10;119(3):e2114886119. doi: 10.1073/pnas.2114886119 (PMC8784102; doi:10.1073/pnas.2114886119)
Supplement: Supplementary File [file pnas.2114886119.sapp.pdf]

Supplementary Information for

Open reading frame correction using splice-switching antisense oligonucleotides for the treatment of cystic fibrosis

Wren E. Michaels[1,2], Cecilia Pena-Rasgado[1], Rusudan Kotaria[1], Robert J. Bridges[1*], and Michelle L. Hastings[1*]

[1] Center for Genetic Diseases, Chicago Medical School, Rosalind Franklin University of Science and Medicine, North Chicago, IL, 60064, United States of America
[2] School of Graduate and Postdoctoral Studies, Rosalind Franklin University of Science and Medicine, North Chicago, IL, 60064, United States of America

* To whom correspondence should be addressed. Tel: 1-847-578-8517; Fax: 1-847-578-3253; Email: michelle.hastings@rosalindfranklin.edu. Correspondence may also be addressed to robert.bridges@rosalindfranklin.edu.

This PDF file includes:

Figures S1 to S3 (not allowed for Brief Reports)
Tables S1
SI References

A

ASO-23A				
Gene	location	intron/exon length	position (nucleotides)	Basepairs
IL1RAPL1	Intron 2	493512nt	123707 from 3' splice site	16
SGSM2	Intron 1	5799nt	1474 from 3' splice site	16
FHIT	Intron 5	522707nt	103109 from 5' splice site	16
PRKX	Intron 3	13177nt	3209 from 3' splice site	16
PHACTR3	Intron 3	7346nt	2242 from 5' splice site	15
EHBP1	Intron 10	68185nt	28107 from 3' splice site	15
PRKCB	Intron 2	152159nt	72531 from 5' splice site	15
JCAD	Intergenic	n/a	47823 upstream of AUG	15
DNAH9	Intron 49	19104nt	4183 from 3' splice site	15
MAOB	Intron 3	35463nt	4009 from 5' splice site	15
SCN10A	Intron 16	1291nt	207 from 5' splice site	15
CACNA1D	Exon 24	88nt	2 from 5' splice site	15
RORA	Intron 1	550366nt	213540 from 5' splice site	15
SETX	Intron 10	14467nt	4347 from 3' splice site	15

ASO-23B				
Gene	location	intron/exon length	position (nucleotides)	Basepairs
FAT4	Intron 6	17622nt	3023 from 3' splice site	16
PCDH15	Intergenic	n/a	841651 from AUG	15
AMMECR1	Intron 2	47970nt	23098 from 3' splice site	15
EXOC6B	Intron 6	142884nt	1844 from 3' splice site	15
MPDZ	Intron 1	29027nt	11847 from 5' splice site	15
FAM120C	Exon 16	4964nt	1541 from 5' splice site	15
AKT2	Exon/Intron 3	exon:129nt intron:1656nt	-1 from 5' splice site	15
FTO	Intron 5	5978nt	2158nt from 5' splice site	15
RLBP1	Intron 5	1881nt	237 from 5' splice site	15

B

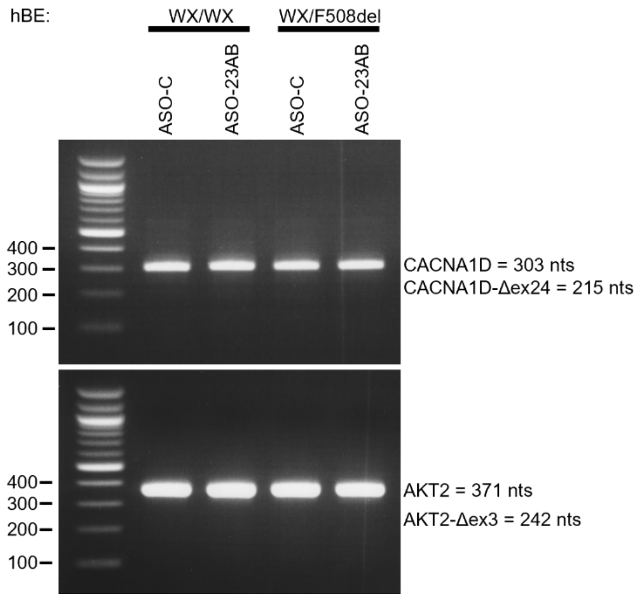

Fig. S1. Off-target analysis of ASO-23A and ASO-23B. (A) Table of results from a BLAST search for human sequences with 15 or more contiguous nucleotides complementary to ASO-23A (top) or ASO-23B (bottom). Targets within exons or near splice sites (within 200 nucleotides) are in red. (B) RT-PCR analysis of RNA from hBE cells homozygous for *W1282X-CFTR* or heterozygous for *W1282X-CFTR* and *F508del-CFTR* treated with ASO-C or ASO-23AB using primers in exons flanking the region with potential ASO binding sites.

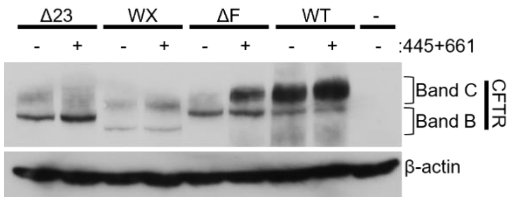

Fig. S2. Immunoblot analysis of CFTR protein in cell lysates analyzed in Figure 1. CFTR protein, Bands C and B, isolated from FRT cells stably transfected with CFTR-Δ23, CFTR-W1282X, CFTR-F508del, CFTR-WT, or empty vector (-) expression plasmids treated with vehicle or VX-445 + VX-661. β-actin was used as a loading control.

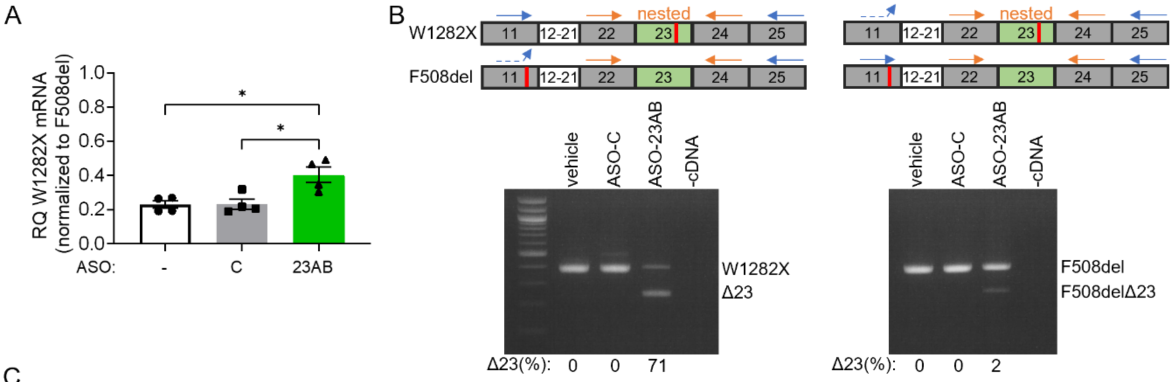

C

CFTR exon 23:

GTGGGCCTCTTGGGAAGAACTGGATCAGGGAAGAGTACTTTGTTATCAGCTTTTTTGAGACTACTGAACACTGAAGGAGAAATCCAGA
TCGATGGTGTGTCTTGGGATTCAATAACTTTGCAACAGTGGAGGAAAGCCTTTGGAGTGATACCACAG

SRSF1 threshold: 1.956	SRSF1 (IgM-BRCA1) threshold: 1.867	SRSF2 threshold: 2.383	SRSF5 threshold: 2.67	SRSF6 threshold: 2.676
Position/Site/Score	Position/Site/Score	Position/Site/Score	Position/Site/Score	Position/Site/Score
57 GAGACTA 2.64552	71 CTGAAGG 2.2387	4 GGCCTCTT 3.43225	58 AGACTAC 2.79705	96 TGTGTC 4.58418
71 CTGAAGG 1.96982	124 CAGTGGA 3.15115	5 GCCTCTTG 2.45572	61 CTACTGA 3.02819	119 TGCAAC 2.92201
124 CAGTGGA 3.28787	127 TGGAGGA 2.00488	59 GACTACTG 5.38755	123 ACAGTGG 3.30474	
127 TGGAGGA 2.34103		66 GAACACTG 4.60771		
		87 GATCGATG 3.3474		
		105 GGATTCAA 2.97789		
		149 TACCACAG 2.54564		

CFTR W1282X:

GTGGGCCTCTTGGGAAGAACTGGATCAGGGAAGAGTACTTTGTTATCAGCTTTTTTGAGACTACTGAACACTGAAGGAGAAATCCAGA
TCGATGGTGTGTCTTGGGATTCAATAACTTTGCAACAGTGAAGGAAAGCCTTTGGAGTGATACCACAG

SRSF1 threshold: 1.956	SRSF1 (IgM-BRCA1) threshold: 1.867	SRSF2 threshold: 2.383	SRSF5 threshold: 2.67	SRSF6 threshold: 2.676
Position/Site/Score	Position/Site/Score	Position/Site/Score	Position/Site/Score	Position/Site/Score
57 GAGACTA 2.64552	71 CTGAAGG 2.2387	4 GGCCTCTT 3.43225	58 AGACTAC 2.79705	96 TGTGTC 4.58418
71 CTGAAGG 1.96982		5 GCCTCTTG 2.45572	61 CTACTGA 3.02819	119 TGCAAC 2.92201
		59 GACTACTG 5.38755		
		66 GAACACTG 4.60771		
		87 GATCGATG 3.3474		
		105 GGATTCAA 2.97789		
		149 TACCACAG 2.54564		

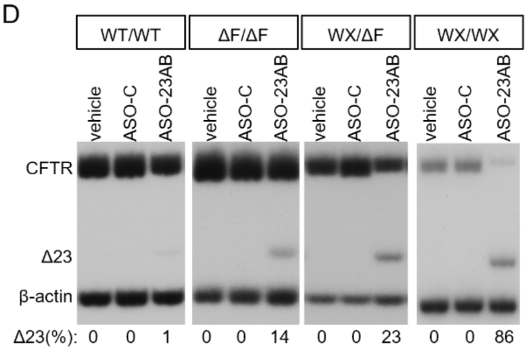

Fig. S3. ASO-induced exon 23 skipping has partial allele specificity for *CFTR-W1282X*. (A) RT-qPCR analyses of RNA isolated from compound heterozygous hBE cells shown in Figure 5 F&H. Total *CFTR* RNA from the *CFTR-W1282X* allele was normalized to RNA from the F508del allele for each treatment group. Error bars are ±SEM. One-way ANOVA; Tukey's multiple comparison test, *p<0.05. N=2. (B) Allele specific RT-PCR analysis of ASO-induced exon 23 skipping in hBE cells from the heterozygous donor shown in Figure 5A. Allele specific transcripts were amplified using primers in exons 11 and 25 (primers: 11WT-25 [W1282X] or 11ΔF-25 [F508del]) (blue). Exon 23 skipping from each allele was analyzed using nested exon 22-24 primers (orange). Exon 23 skipping was quantified (% of total) and is shown below each lane. Diagrams of primer annealing sites are shown. Hashed lines indicate mismatch with indicated amplicon. (C) Comparison of calculated SR protein binding sites

[ESEfinder, Cold Spring Harbor Laboratory, (1)] between WT exon 23 (top) and exon 23 containing the CFTR-W1282X mutation (bottom). Differences are indicated in red. (D) RT-PCR analysis of CFTR exon 23 skipping in hBE cells from various donors treated with vehicle, ASO-C, or ASO-23AB (320 µM). The genotype of each donor is indicated. Exon 23 skipping is indicated below each lane. β-actin is a control for RNA expression.

ASOs	Sequence (5’-3’)
ASO-23A	CTAAGTCCTTTTGCTCACCTGTGGT
ASO-23B	AAGTTATTGAATCCCAAGACACACC
ASO-23C	AGCTGATAACAAAGTACTCTTCCCT
ASO-23D	ATCCAGTTCTTCCCAAGAGGCCCAC
ASO-C	CCTCTTACCTCAGTTACAATTTATA
**Primers**	**Sequence (5’-3’)**
1:HCAI-CFTRdel23R	GCGCTGGCCGGGGCTGAT
2:HCAI-CFTRdel23F	AAGGTGTTCATCTTCAGCGGCACCTTC
3: HCAI-CFTRWXF	TGCAGCAGTGACGCAAGGCCT
4: HCAI-CFTRWXR	GGGTGATGCTGTCCCAGC
5: hCFTR-ex11ΔFF	GCCTGGCACCATTAAAGAAAATATCATTGG
6: hCFTR-ex11F	GCCTGGCACCATTAAAGAAAATATCATCTT
7: hCFTR-ex14R	TCCAGGAGACAGGAGCATCT
8: hCFTR-ex22F	CCAAACCATACAAGAAT
9: hCFTR-ex24R	GATCACTCCACTGTTCAT
10: hCFTR-ex25R	GTTCTATCACAGATCTGAG
11: qhCFTR-ex11WTF	TGGCACCATTAAAGAAAATATCATCTT
12: qhCFTR-ex12WTR	CTCAGTGTGATTCCACCTTCTC
13: qhCFTR-ex11ΔFF	GGCACCATTAAAGAAAATATCATTGG
14: qhCFTR-ex12ΔFR	CTCAGTGTGATTCCACCTTCT
15: hβ-actinFor	AAAGACCTGTACGCCAACAC
16: hβ-actinRev	GTCATACTCCTGCTTGCTGAT
17: qhHPRT1For	GCGATGTCAATAGGACTCCAG
18: qhHPRT1Rev	TTGTTGTAGGATATGCCCTTGA
**Probes**	**Sequence (5’-3’)**
hCFTR-DF508	/56-FAM/ACAGAAGCG/ZEN/TCATCAAAGCATGCC/3IABkFQ/
hCFTR-F508	/5.6-FAM/ACAGAAGCG/ZEN/TCATCAAAGCATGCC/3IABkFQ/
hHPRT1	/5HEX/AGCCTAAGA/ZEN/TGAGAGTTCAAGTTGAGTTTGG/3IABkFQ/

Table S1. Splice-switching antisense oligonucleotide, primers, and probes used in the manuscript

SI References

1. L,C., J,W., Z,Z., MQ,Z. and AR,K. (2003) ESEfinder: A web resource to identify exonic splicing enhancers. *Nucleic Acids Res.*, **31**, 3568–3571.
